# Microarray Analysis of Rat Pancreas Reveals Altered Expression of Alox15 and Regenerating Islet-Derived Genes in Response to Iron Deficiency and Overload

**DOI:** 10.1371/journal.pone.0086019

**Published:** 2014-01-21

**Authors:** Richard Coffey, Hyeyoung Nam, Mitchell D. Knutson

**Affiliations:** Food Science and Human Nutrition Department, University of Florida, Gainesville, Florida, United States of America; Lady Davis Institute for Medical Research/McGill University, Canada

## Abstract

It is well known that iron overload can result in pancreatic iron deposition, beta-cell destruction, and diabetes in humans. Recent studies in animals have extended the link between iron status and pancreatic function by showing that iron depletion confers protection against beta-cell dysfunction and diabetes. The aim of the present study was to identify genes in the pancreas that are differentially expressed in response to iron deficiency or overload. Weanling male Sprague-Dawley rats (n = 6/group) were fed iron-deficient, iron-adequate, or iron-overloaded diets for 3 weeks to alter their iron status. Total RNA was isolated from the pancreases and pooled within each group for microarray analyses in which gene expression levels were compared to those in iron-adequate controls. In iron-deficient pancreas, a total of 66 genes were found to be differentially regulated (10 up, 56 down), whereas in iron-overloaded pancreas, 164 genes were affected (82 up, 82 down). The most up-regulated transcript in iron-deficient pancreas was arachidonate 15-lipoxygenase (Alox15), which has been implicated in the development of diabetes. In iron-overloaded pancreas, the most upregulated transcripts were Reg1a, Reg3a, and Reg3b belonging to the regenerating islet-derived gene (Reg) family. Reg expression has been observed in response to pancreatic stress and is thought to facilitate pancreatic regeneration. Subsequent qRT-PCR validation indicated that Alox15 mRNA levels were 4 times higher in iron-deficient than in iron-adequate pancreas and that Reg1a, Reg3a, and Reg3b mRNA levels were 17–36 times higher in iron-overloaded pancreas. The elevated Alox15 mRNA levels in iron-deficient pancreas were associated with 8-fold higher levels of Alox15 protein as indicated by Western blotting. Overall, these data raise the possibility that Reg expression may serve as a biomarker for iron-related pancreatic stress, and that iron deficiency may adversely affect the risk of developing diabetes through up-regulation of Alox15.

## Introduction

The association between excess iron and pancreatic dysfunction has long been observed in the iron overload disorder hereditary hemochromatosis [1]. Patients with hemochromatosis have a higher prevalence of diabetes, decreased insulin secretory capacity, and impaired glucose tolerance relative to the normal population [2]. The *Hfe* knockout mouse, the animal model of hemochromatosis, also displays alterations in pancreatic function, including decreased insulin secretory capacity [3]. In humans, insulin secretory capacity and glucose tolerance improves after iron stores are normalized by phlebotomy, suggesting that tissue iron levels are an important determinant of insulin action [4]. Consistent with this idea are animal studies showing that a decrease in iron stores (in response to phlebotomy or a low-iron diet) can increase insulin secretion and pancreatic insulin levels [5], [6]. However, iron depletion to the point of iron deficiency and anemia has been shown to negatively affect glucose homeostasis by increasing blood glucose concentrations [7].

The effects of iron overload and deficiency on glucose homeostasis are likely mediated, at least in part, by iron-related changes in the expression of genes involved in glucose metabolism. For example, iron deficiency has been reported to be associated with higher levels of rate-limiting gluconeogenic enzymes in rat liver [8] and iron-loaded *Hfe* knockout mice display increased glucose uptake by isolated soleus muscle and decreased glucose oxidation by isolated hepatic mitochondria [9], [10]. Little information, however, exists regarding iron-related gene expression in the pancreas. Given that the pancreas hormonally controls whole-body glucose homeostasis, the aim of the present study was to examine global changes in pancreatic gene expression in response to iron deficiency and overload. Identification of pancreatic genes that are regulated by iron status may offer insight not only into how iron status perturbs glucose homeostasis, but also how iron overload may contribute to beta-cell destruction and diabetes.

## Materials and Methods

### Animals and Diets

Weanling male Sprague-Dawley rats (Charles River Laboratories) were randomized (n = 6/group) to receive either iron-deficient (FeD), iron-adequate (FeA), or iron-overloaded (FeO) diets. Purified diets were prepared according to the AIN-93G formulation, but with no added iron (FeD), 35 mg/kg ferric citrate (FeA), or 2% carbonyl iron (Sigma-Aldrich) (FeO). Iron contents of the diets, as determined by inductively coupled plasma mass spectroscopy (ICP-MS), were 5 ppm (FeD), 36 ppm (FeA), and 20,275 ppm (FeO). Diets were also modified to contain Avicel® microcrystalline cellulose instead of cellulose (to minimize contaminant iron) and 20% sucrose instead of 10% sucrose (while reducing the amount of cornstarch accordingly) [11]. The amount of sucrose was increased in order to make the iron-loaded diet more palatable. After 3 weeks of feeding, overnight-fasted rats were sacrificed by exsanguination from the descending aorta. Blood was collected into heparinized syringes and then centrifuged to obtain plasma. Pancreases were quickly harvested, immediately frozen in liquid nitrogen, and maintained at −80°C for subsequent analyses. Animal experiments were approved by the Institutional Animal Care and Use Committee at the University of Florida (Protocol # 201101613).

### Iron Status Parameters and Blood Glucose Concentrations

Hemoglobin and liver non-heme iron were measured as described previously [11]. Blood glucose concentrations were determined in freshly collected heparinized blood by using a handheld glucometer (Accu-Chek)

### Pancreatic Mineral Concentrations

Tissues were dried overnight in a 75°C oven and then digested overnight in approximately 10× its mass of nitric acid. The digested samples were diluted with water to 100× the dried tissue mass. Elemental analysis for iron, zinc, manganese, copper, and cobalt was performed according to the method of Wahlen et al. [12] using an Agilent 7500ce Inductively Coupled Plasma–Mass Spectrometer (ICP/MS). Analyses were performed by the Michigan State University Diagnostic Center for Population and Animal Health.

### RNA Isolation and Assessment of RNA Integrity

Frozen pancreas samples were submerged in liquid nitrogen and finely ground by using a mortar and pestle. After grinding, total RNA was isolated by using the RNeasy Mini Kit (Qiagen) following the manufacturer's protocol. Integrity of isolated RNA was confirmed by denaturing agarose gel electrophoresis followed by visualization of 18S and 28S ribosomal RNA bands. Prior to microarray analysis, RNA integrity was additionally assessed by using the Agilent 2100 Bioanalyzer and RNA 6000 Nano Kit (Agilent).

### Microarray Analysis

RNA samples (n = 6) from each dietary group were pooled and analyzed by using a Rat GE 4x44K v3 Microarray (Agilent). Both FeD and FeO pooled cDNA samples were individually compared with FeA in duplicate measurements. The assignment of fluorescent Cy3 or Cy5 dye to each comparison group was alternated between the duplicates. To identify differential gene expression, values of signal intensity were log_2_ transformed and normalized before the Student's t-test was performed for probe-specific comparisons. Genes showing a statistically significant *(P*<0.05) log_2_-transformed fold change of at least ±2 were analyzed to identify functional biological categories by using the Database for Annotation, Visualization and Integrated Discovery (DAVID) [13]. Microarray analysis was conducted at the Interdisciplinary Center for Biotechnology at the University of Florida. The microarray data discussed herein have been deposited in NCBI's Gene Expression Omnibus [14] and are accessible through GEO Series accession number GSE44699 (http://www.ncbi.nlm.nih.gov/geo/query/acc.cgi?acc=GSE44699).

### Relative mRNA Quantification

cDNA was synthesized from total RNA by using the High Capacity cDNA Reverse Transcription Kit (Applied Biosystems). Specific exon-spanning primers for genes of interest were generated (Table S1) and confirmed for specificity by using the NCBI Basic Local Alignment and Search Tool (BLAST) [15]. Quantitative reverse transcriptase polymerase chain reaction (qRT-PCR) was performed by using *Power* SYBR Green PCR Mastermix (Applied Biosystems) and an Applied Biosystems 7300 Real-Time PCR System. Dissociation curve analysis of PCR products revealed single products and all PCR amplification efficiencies were 100±10%. Quantitation of mRNA was determined by comparison to standard curves generated by four 10-fold serial dilutions of standard cDNA. Transcript levels were normalized to those of cyclophilin B (peptidylprolyl isomerase B, PPIB).

### Western Blotting

Pancreas samples were homogenized in ice-cold buffer containing 0.05 M Tris-HCl (pH 7.4), 0.05 M NaCl, 0.001 M EDTA, 0.25% Tween-20, and Complete, Mini Protease Inhibitor Cocktail (Roche). Tissue homogenates were clarified by centrifugation at 10,000×*g* for 10 minutes at 4°C, followed by sonication of the supernatant. Protein concentrations were determined by using the *RC DC* Protein Assay Kit (Bio-Rad). Proteins were mixed with Laemmli buffer, incubated at 70°C for 10 minutes, and then electrophoretically separated by sodium dodecyl sulfate-polyacrylamide gel electrophoresis (SDS-PAGE) on a 7.5% gel. Separated proteins were transferred to a polyvinyl difluoride (PVDF) membrane (Bio-Rad), and incubated in blocking buffer (5% nonfat dry milk in Tris-buffered saline (TBS)-Tween 20 (TBS-T)) for 1 hour. The blot was then incubated with rabbit anti-rat Alox15 antibody (kindly provided by Dr. James F. Collins, University of Florida), 1∶8000 dilution for 2 hours. After washing with TBS-T, the blot was incubated with horseradish peroxidase (HRP)-conjugated donkey anti-rabbit IgG secondary antibody (Amersham Biosciences), 1∶10,000 dilution for 45 minutes. After washing with TBS-T and TBS, antibody binding was observed by using enhanced chemiluminescence (SuperSignal West Pico, Pierce) and the Fluorchem E imaging system (ProteinSimple). To indicate lane loading, the blot was stripped and reprobed with a mouse anti-α-tubulin antibody (Sigma) at a 1∶5000 dilution, followed by an HRP-conjugated goat anti-mouse IgG secondary antibody (Santa Cruz) at a 1∶10,000 dilution. Densitometry was performed by using AlphaView software (ProteinSimple).

### Statistical Analysis

Data were analyzed for statistical significance by using one-way ANOVA and Tukey's multiple comparison post-hoc test (GraphPad Prism). Unequal variance between groups was accounted for by log transformation, where applicable, to normalize variance before statistical analysis.

## Results

### Body Weight, Iron Status, and Blood Glucose Concentrations

After 3 weeks of feeding the experimental diets, body weights were significantly lower in the FeD and FeO groups relative to FeA controls, but did not differ between FeD and FeO animals (Table 1). Liver non-heme iron concentrations, an indicator of body iron stores, confirmed that rats fed the FeD diet became iron deficient whereas rats fed the FeO diet became iron overloaded. In FeO animals, liver non-heme iron concentrations were nearly 40 times higher than controls. FeD rats also became anemic with hemoglobin levels that were 41% lower than normal (Table 1). Blood glucose concentrations were elevated in FeD rats compared with FeA controls, whereas those in FeO animals did not differ from controls (Table 1).

**Table 1:** Body weight, iron status, and blood glucose concentration of rats.

Group*	Body weight (g)	Liver non-heme iron (μg/g)	Hemoglobin (g/dL)	Glucose (mg/dL)
FeD	193.3±20.2^a^	3.5±3.6^a^	7.5±2.2^a^	154.5±39.0^b^
FeA	224.3±11.1^b^	25.4±17.7^b^	12.8±0.4^b^	99.0±16.0^a^
FeO	170.3±23.6^a^	980.6±310.2^c^	13.6±0.6^b^	117.7±18.5^a^

* FeD, iron deficient.; FeA, iron adequate; FeO, iron overloaded. Values represent means ± SD, n = 6.

Means without a common superscript are significantly different *P*<0.05.

### Pancreatic Mineral Concentrations

In FeO rats, pancreatic iron concentrations were 155% higher than those in FeA animals, whereas in FeD rats, iron concentrations were 40% lower than controls (Table 2). Given that iron deficiency and overload can affect tissue concentrations of other trace minerals [11], we measured pancreatic concentrations of zinc, manganese, copper, and cobalt (Table 2). Pancreatic zinc concentrations were found to be 26% higher in FeD rats, and copper concentrations were 74% lower in FeO rats, when compared with FeA controls. By contrast, pancreatic manganese and cobalt concentrations did not differ among groups. It should be noted that the concentrations of zinc, manganese, copper, and cobalt did not differ among the experimental diets (data not shown).

**Table 2:** Pancreatic mineral concentrations.

Group	Iron	Zinc	Manganese	Copper	Cobalt
FeD	38.2±5.7^a^	107.5±18.0^b^	8.7±1.9	4.7±1.0^b^	0.05±0.02
FeA	63.7±14.3^b^	85.0±16.0^a^	6.2±1.9	3.8±0.7^b^	0.03±0.01
FeO	162.7±59.5^c^	75.2±8.5^a^	7.0±1.2	1.0±0.0^a^	0.04±0.01

Mineral concentrations (μg/g dry weight) were measured by using ICP-MS. Values represent means ± SD, n = 6 Means without a common superscript are significantly different *P*<0.05.

### Identification and Classification of Differentially Expressed Genes by Microarray Analysis

**Figure 1:**
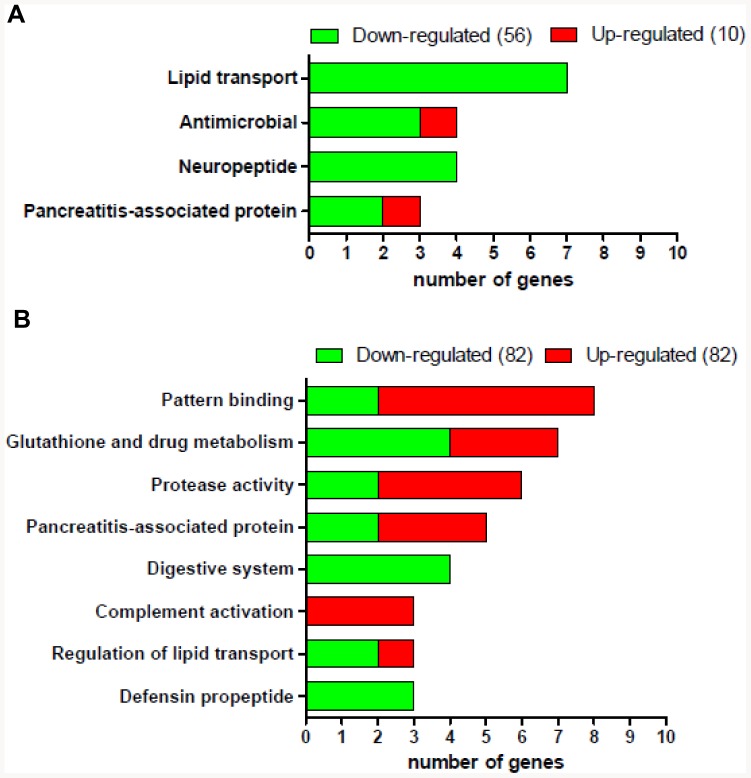
Functional classification of pancreatic genes up- or down-regulated in response to iron deficiency and iron overload. Microarray analysis identified a total of 66 differentially expressed genes in response to iron deficiency (Panel A) and 164 genes in response to iron overload (Panel B). Genes were then subjected to DAVID analysis to identify functional categories. (A) Functional gene categories identified in iron-deficient pancreas and the number of genes in each category. (B) Functional gene categories identified in iron-overloaded pancreas and the number of genes in each category.

Microarray analysis was used to identify candidate genes that are differentially expressed in FeD and FeO pancreas, especially those that may influence the risk for diabetes. Using a log_2_ fold change of ±2 and *P*<0.05 as a cutoff, we identified a total of 230 genes as differentially expressed in FeD and FeO pancreas relative to FeA pancreas. In FeD pancreas 66 genes were differentially expressed (56 down-regulated and 10 up-regulated) (Figure 1). In FeO pancreas 164 genes were differentially expressed (82 down-regulated and 82 up-regulated). The differentially expressed genes were analyzed by using DAVID bioinformatics resources to identify gene ontology categories. In FeD pancreas, the category with the highest number of genes was “lipid transport” (7 genes), followed by “antimicrobial” (4 genes), “neuropeptide” (4 genes), and “pancreatitis-associated protein” (3 genes) (Figure 1A). All but two of the genes in these categories were down-regulated in FeD pancreas. By contrast, in FeO pancreas, most gene ontology categories were enriched with up-regulated genes (Figure 1B). For example, 6 of 8 genes were up-regulated in the “pattern binding” category in FeO pancreas. Of note, the gene ontology category “pancreatitis-associated protein” was identified in both FeD and FeO pancreas. Lists of the genes in each category along with fold change are provided in Table S2 and Table S3. The top 10 most up-regulated and down-regulated genes in FeD and FeO pancreas, ordered by mean magnitude change (*P*<0.05), are shown in Table 3 and Table 4. The genes listed in Table S2, Table S3, Table 3, and Table 4 were surveyed in the literature to identify those with reported associations with diabetes and/or glucose homeostasis, and several of these were subsequently selected for validation by qRT-PCR of individual rat samples (n = 6 group). It should be noted that, although we altered the iron status of the animals and the pancreases, the microarray did not detect changes in the expression of any genes commonly known to be related to iron homeostasis (e.g. *Slc11a2, Slc40a1, Slc39a14, Cybrd1, Heph, Trfr1, Trfr2, Hfe, Hamp*). QRT-PCR analyses of *Slc11a2* and *Slc39a14* confirmed no differences in the expression of these genes (data not shown). However, we did find that *Trfr1* (transferrin receptor) mRNA levels were 58% higher (*P*<0.05) in the FeD pancreases relative to the FeA controls (data not shown), consistent with the known regulation of TfR1 in iron-deficient conditions.

**Table 3:** Top 10 most up-regulated and down-regulated genes in FeD pancreas.

Up-regulated genes	Symbol	Accession	Fold change^a^
arachidonate 15-lipoxygenase	*Alox15*	NM_031010	4.1
L-threonine dehydrogenase	*Tdh*	NM_001106044	3.3
RT1 class I, locus CE5	*RT1-CE5*	NM_001008843	3.3
S100 calcium binding protein A9*	*S100a9*	NM_053587	2.7
transient receptor potential cation channel, subfamily C, member 3	*Trpc3*	NM_021771	2.4
vascular endothelial growth factor B	*Vegfb*	NM_053549	2.4
regenerating islet-derived 1 alpha*	*Reg1a*	NM_012641	2.2
secretoglobin, family 2A, member 1	*Scgb2a1*	NM_080770	2.2
potassium intermediate/small conductance Ca-activated channel, subfamily N, member 1	*Kcnn1*	NM_019313	2.0
alanine-glyoxylate aminotransferase 2	*Agxt2*	NM_031835	1.9
Down-regulated genes			
fatty acid binding protein 1, liver*	*Fabp1*	NM_012556	−6.1
fatty acid binding protein 2, intestinal*	*Fabp2*	NM_013068	−6.0
LOC494499 protein	*LOC494499*	NM_001010921	−5.0
proline-rich acidic protein 1	*Prap1*	NM_031669	−5.0
s100 calcium binding protein G	*S100g*	NM_012521	−4.9
monoacylglycerol O-acyltransferase 2*	*Mogat2*	NM_001109436	−4.9
apolipoprotein A-I*	*Apoa1*	NM_012738	−4.6
cAMP responsive element binding protein 3-like 3	*Creb3l3*	NM_001012115	−4.5
similar to carboxylesterase 5	*LOC679368*	XM_001056053	−4.3
carboxylesterase 5-like	LOC688542	XR_086144	−4.2

a Fold change log_2_ relative to iron-adequate rat pancreas.

* In gene ontology category in Figure 1 and Table S2.

**Table 4:** Top 10 most up-regulated and down-regulated genes in FeO pancreas.

Up-regulated genes	Symbol	Accession	Fold change^a^
regenerating islet-derived 3 alpha*	*Reg3a*	NM_172077	4.8
regenerating islet-derived 3 beta*	*Reg3b*	NM_053289	4.3
extracellular proteinase inhibitor	*Expi*	NM_133537	4.3
regenerating islet-derived 1 alpha*	*Reg1a*	NM_012641	4.2
Prepronociceptin	*Pnoc*	NM_013007	3.8
beta-galactosidase-like protein	*Bin2a*	NM_001009524	3.6
calmodulin-like 3	*Calml3*	NM_001012054	3.5
vascular endothelial growth factor B*	*Vegfb*	NM_053549	3.5
phospholipase A2, group IIA*	*Pla2g2a*	NM_031598	3.5
upper zone of growth plate and cartilage matrix associated	*Ucma*	NM_001106121	3.2
Down-regulated genes			
similar to Robo-1	*LOC691352*	NM_001109638	−8.7
fatty acid binding protein 2, intestinal*	*Fabp2*	NM_013068	−7.0
fatty acid binding protein 1, liver*	*Fabp1*	NM_012556	−7.0
proline-rich acidic protein 1	*Prap1*	NM_031669	−6.6
lectin, galactoside-binding, soluble, 4	*Lgals4*	NM_012975	−6.0
apolipoprotein A-I *	*Apoa1*	NM_012738	−5.8
hydroxysteroid (17-beta) dehydrogenase 2	*Hsd17b2*	NM_024391	−5.6
S100 calcium binding protein G	*S100g*	NM_012521	−5.6
LOC494499 protein*	*LOC494499*	NM_001010921	−5.4
hypothetical protein LOC691259	*LOC691259*	NM_001109632	−5.2

a Fold change log_2_ relative to iron-adequate rat pancreas.

* In gene ontology category in Figure 1 and Table S3.

### Confirmation of Up-Regulation of Alox15 Expression by QRT-PCR and Western Blotting

**Figure 2:**
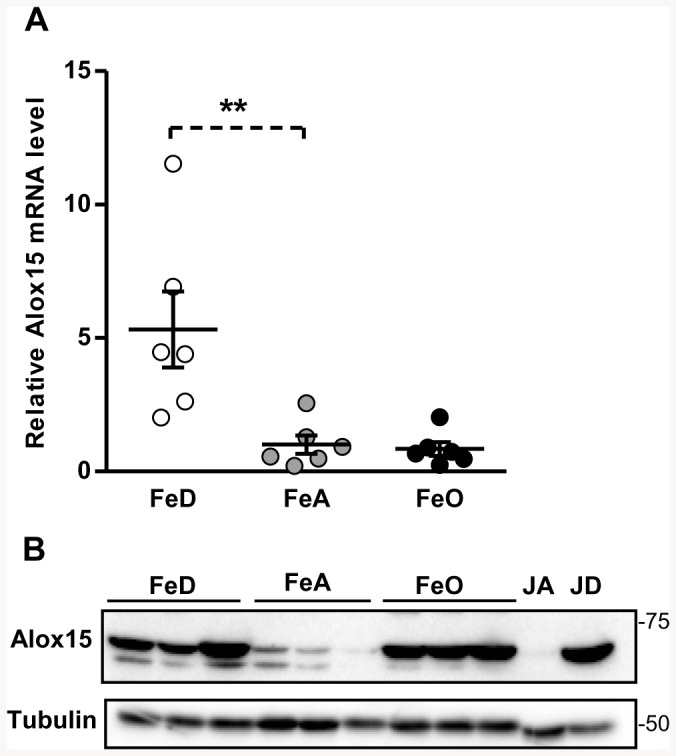
Effect of iron deficiency and overload on rat pancreatic Alox15 expression. A) Total RNA was isolated from rat pancreas and the relative transcript abundance of Alox15 was measured by using qRT-PCR. Transcript abundances were normalized to the housekeeping transcript cyclophilin B and are expressed relative to the FeA group mean (set to 1). B) Immunoblot analysis of Alox15 from a representative sample of FeD, FeA, and FeO rats. Jejunum from iron-adequate (JA) and iron-deficient (JD) rats were analyzed in parallel to serve as negative and positive controls respectively for immunodetection of Alox15. The blot was stripped and reprobed for tubulin to indicate protein loading among lanes. Values are expressed as the mean ± SEM, n = 6. Asterisks indicate a significant difference relative to FeA controls, **P<0.01.

According to the microarray analysis, the most up-regulated gene in FeD pancreas was *Alox15* (arachidonate 15-lipoxygenase) (Table 3). Alox15 catalyzes the oxidation of polyunsaturated fatty acids, such as arachidonic acid, during the formation of inflammatory mediators and has been linked to the development of type 1 diabetes [16], [17]. QRT-PCR analysis confirmed the up-regulation of Alox15 mRNA levels in FeD pancreas (Fig. 2A), and Western blot analysis revealed higher Alox15 protein levels in FeD pancreas (Fig. 2B). Alox15 protein levels were also found to be higher in FeO pancreas compared with FeA controls despite no increase in Alox15 mRNA levels. As Western blotting controls for rat Alox15, jejunum samples from iron-adequate (JA) and iron-deficient (JD) rats were analyzed in parallel with the rat pancreas samples. Consistent with a previous study by Collins et al. [18], Alox15 was detected at approximately 70 kDa and was markedly up-regulated in iron-deficient jejunum (JD) (Fig. 2B). Densitometric analysis of the Western blots indicated that Alox15 protein levels were approximately 8- and 9-fold higher (*P*<0.001) in FeD and FeO pancreas, respectively, compared with FeA controls (n = 6/group; data not shown).

### Confirmation of Reg Family mRNA Levels by QRT-PCR

**Figure 3:**
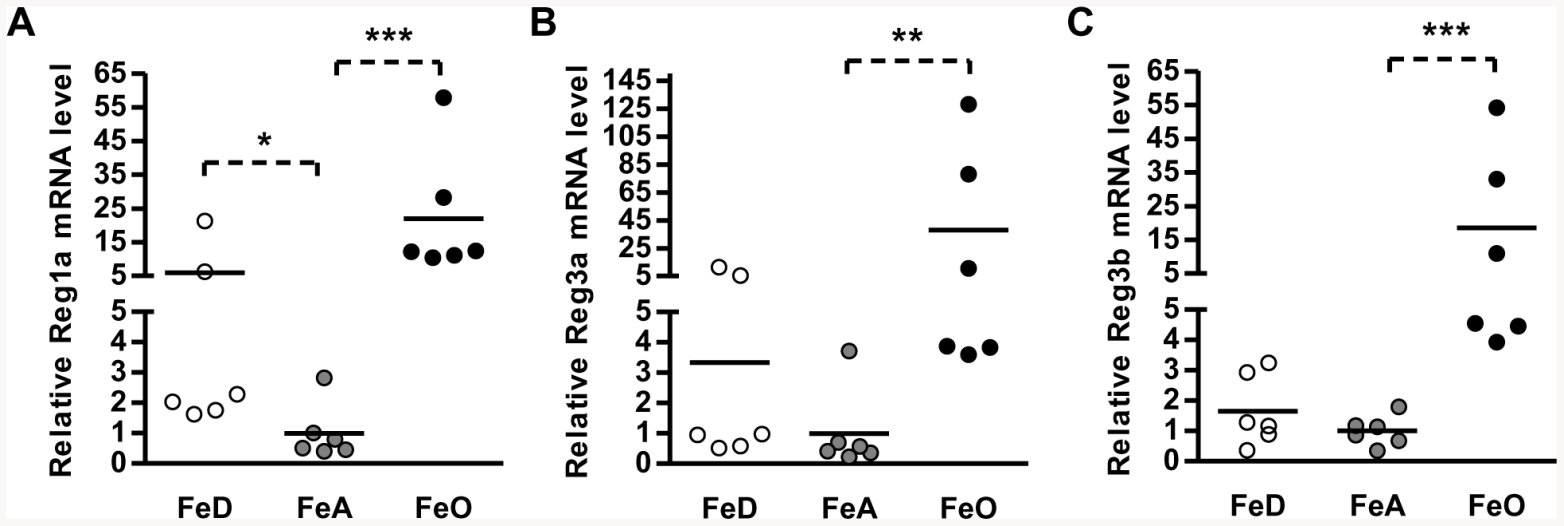
Effect of iron deficiency and overload on the expression of pancreatic Reg family genes. Total RNA was isolated from rat pancreas and the relative transcript abundances of Reg family genes were determined by qRT-PCR. Transcript abundances were normalized to levels of cyclophilin B and are expressed relative to the FeA group mean (set to 1). Statistical significance was determined by one-way ANOVA. Asterisks indicate a significant difference relative to FeA controls *P<0.05, **P<0.01, ***P<0.001.

Of the genes showing positive regulation during FeO, the most elevated belonged to the *Reg* family of regenerating islet-derived genes (Table 4). The genes of the *Reg* family, most notably *Reg1a*, have been linked to pancreatic regeneration as well as cellular growth and survival during oxidative stress [19]–[22]. Consistent with the microarray data, qRT-PCR analysis revealed that mRNA levels of these genes were up-regulated in FeO pancreas. Mean mRNA levels of Reg1a, Reg3a, and Reg3b were found to be 21, 37, and 18 times higher, respectively, in FeO pancreas than FeA controls (Fig. 3). Also consistent with the microarray, qRT-PCR analysis found that Reg1a mRNA levels were up-regulated in FeD pancreas. Reg mRNA levels varied considerably among rats, particularly in the FeO and FeD groups in which two or three values were notably higher than the others. Repeated analyses confirmed that the high values do not represent analytical artifacts. In the FeD, FeA, and FeO groups, the high values for Reg1a and Reg3a (but not Reg3b) mRNA are from the same animals, suggesting that Reg1a and Reg3a are up-regulated in parallel. It would be informative to know whether these marked differences in Reg mRNA levels are associated with changes in protein levels, however, we have been unable to locate suitable antibodies. To our knowledge, no anti-rat Reg1a antibodies are currently available, and the one anti-rat Reg3a antibody that we tried (anti-rat Reg3a, R & D Systems, catalogue #AF1745) did not provide reliable results (data not shown).

### Discrepancies between microarray and qRT-PCR analysis results

According to the microarray, both FeD and FeO pancreas showed large down-regulations in the expression of *Fabp1*, *Fabp2*, and *Apoa1* (Tables 3 and 4), which clustered in the gene ontology category of “lipid transport” (Table S3). As all of these genes have been associated with diabetes in the surveyed literature [23]–[25] they were selected for follow-up study. However, the expression levels of these genes in pancreas were found to be below the detection limit of qRT-PCR, similar to previous studies that have failed to detect the expression of these genes in pancreas [26], [27].

## Discussion

Because iron status can affect glucose homeostasis, we sought to identify glucose metabolism-related genes in rat pancreas whose expression might be affected by iron deficiency or overload. Unexpectedly, our microarray data, and subsequent functional enrichment analysis by DAVID, did not identify any changes in the expression of genes known to be involved in glucose metabolism. A limitation to our study is that many of the glucose-responsive genes are found in islet cells [28], which constitute only 1–2% of the mass of the pancreas, and therefore changes in islet-cell gene expression may not be readily detectable in whole pancreas tissue unless large differences exist between treatment groups. The most notable finding from our microarray analyses was the identification of differentially expressed genes that are associated with diabetes and/or pancreatic stress. More specifically, Alox15 was identified as the most up-regulated mRNA in iron deficiency, and Reg family transcripts Reg1a, Reg 3a, and Reg 3b, were found to be markedly up-regulated in iron overload.

*Alox15* encodes arachidonate 15-lipoxygenase, a non-heme iron-containing enzyme that catalyzes the oxygenation of polyunsaturated fatty acids to form inflammatory mediators [29]. Despite the name suggesting 15-lipoxygenase activity, Alox15 in rodents has been demonstrated to function primarily as a 12-lipoxygenase with secondary 15-lipoxygenase function [30]. Therefore the term leukocyte 12-lipoxgenase, as well as the hybrid term 12/15-lipoxygenase, is often used in reference towards Alox15. A link between iron deficiency and Alox15 was first reported in a microarray study by Collins et al. [18], who identified Alox15 as the most strongly induced gene in the intestine of iron-deficient rats. Alox15 has also been identified as the most highly induced gene in microarray studies of iron-deficient rat liver [8] and brain [31]. Similar to Collins et al. [18], we found that elevated Alox15 mRNA levels were associated with higher Alox15 protein levels. The marked up-regulation of Alox15 mRNA expression in iron-deficient pancreas may be related to hypoxia due to iron-deficiency anemia in these animals. Hypoxia in rat pulmonary smooth muscle cells has been reported to induce Alox15 in a process that involves the transcription factor HIF-1α [32]. Interestingly, the Alox15 promoter region does not contain hypoxia response elements (HREs) [33], suggesting that HIF-1α acts indirectly to regulate Alox15 expression. Inspection of the *Alox15* promoter sequence also failed to identify other elements (e.g. antioxidant responsive element, ARE, or a Maf recognition element, MARE) that have been associated with transcriptional activation of other iron-related proteins [34], [35]. Protein levels of Alox15 were also found to be elevated in iron-loaded rat pancreas, despite no up-regulation of Alox15 mRNA levels, suggesting post-transcriptional regulation under iron-overload conditions. Although previous studies have reported possible post-transcriptional regulation of Alox15 [36], our data are the first to suggest its post-transcriptional regulation by iron loading. How this regulation may occur will require further investigation. Notably, Alox15 mRNA does not contain an identifiable iron-response element (IRE), which is commonly associated with mRNAs that are post-transcriptionally regulated by iron [37].

In the pancreas, Alox15 is present in beta cells [38] where it appears to play a role in the pathogenesis of diabetes. Genetic deletion of a locus containing *Alox15* has been shown to protect nonobese diabetic (NOD) mice from developing autoimmune diabetes, with knockout mice exhibiting superior islet mass and glucose tolerance [16]. Recent experiments using siRNA against Alox15 provide evidence that diminished Alox15 levels are responsible for the protective phenotype [17]. Resistance to the development of a diabetic phenotype induced via streptozotocin was also observed in mice lacking *Alox15* [39]. It has been proposed that Alox15 contributes to the development of diabetes via its ability to catalyze the formation of inflammatory mediators such as 12-HETE (hydroxyeicosatetraenoic acid), which causes beta-cell dysfunction and death [38], [40], [41], recently linked to excessive production of reactive oxygen species [42]. Our observation that iron deficiency causes a marked elevation in Alox15 mRNA and protein levels in the pancreas raises the possibility that iron deficiency—in addition to iron overload—may increase the risk of developing diabetes through up-regulation of Alox15. Such a possibility appears opposite to recent studies showing a protective effect of iron restriction on diabetes risk. For example, Cooksey et al. [5] observed that an iron-restricted diet enhanced beta-cell function and insulin sensitivity in the *ob/ob* mouse model of type 2 diabetes. Similarly, Minamiyama et al. [6] found that feeding an iron-restricted diet to type 2 diabetic Otsuka Long-Evans Tokushima fatty (OLETF) rats normalized plasma insulin levels. It should be noted, however, that in the study by Cooksey et al. [5], iron-restriction did not result in iron deficiency or anemia in contrast to our study.

Although it is well known that individuals with iron overload are susceptible to developing diabetes [1], the molecular mechanisms involved remain poorly understood. Our observation that iron-overloaded rats have highly elevated Alox15 protein levels in the pancreas suggests that Alox15 may contribute to beta-cell loss and beta-cell dysfunction in iron overload. Indeed, the pancreases of iron-loaded rats appear to be under stress as indicated by the elevated expression of the regenerating islet-derived gene family members *Reg1a*, *Reg3a*, *and Reg3b*. As indicated by their name, Reg genes were first identified by their strong induction in regenerating pancreatic islets in response to stress/damage [43]. Reg1a is a 165-a.a secreted protein that has been shown to play an important role in beta-cell function in vivo [19]. Disruption of murine *Reg1* (the ortholog of rat *Reg1a*) resulted in decreased proliferative capacity of pancreatic beta cells [44], whereas administration of recombinant rat Reg1a resulted in beta-cell regeneration and reversal of diabetes in rats after surgical resection of 90% of the pancreas [19]. Similar to Reg1, Reg3a and Reg3b have been associated with islet regeneration and protection against diabetes [21], [45]. Reg3 proteins are also known as pancreatitis-associated proteins (PAP) that become highly expressed in acinar cells in response to injury [46]. Our observation of elevated Reg3 expression in iron-loaded rat pancreas is consistent with a previous report of hypotransferrinemic mice, which displayed pancreatic iron loading and markedly elevated expression of Reg3 mRNA [47]. However, in that study, a time course analysis of pancreatic iron loading indicated that Reg3/PAP mRNA became detectable only when pancreatic non-heme iron concentrations had reached levels that were ∼50 times normal. In our study of iron-loaded rats, we found that even modest elevations in pancreatic iron concentrations (2.5 times normal) are associated with enhanced expression of Reg3 mRNA, suggesting that Reg mRNA levels could serve as an early biomarker of iron-related pancreatic stress/damage in rats. The apparent discrepancy in pancreatic iron load required to elicit increased Reg3 expression between mice and rats is likely attributable to interspecies variability. Mice are largely resistant to the degenerative effects of pancreatic iron loading whereas rats exhibit acinar cell degradation, indicative of pancreatic damage, following dietary iron overload [48], [49]. One caveat is that the elevated pancreatic Reg expression in iron-loaded rats could be confounded by the abnormally low (i.e., ∼25% of normal) copper concentrations in these animals. Copper deficiency in rats has been shown to result in pronounced atrophy of the exocrine pancreas [50]. Pancreatic atrophy is observed during pancreatitis, a state which promotes extensive expression of Reg family genes [51]. Also, during copper deficiency islet hyperplasia and beta-cell neogenesis have been documented [52] in line with the islet-regenerating properties of Reg proteins. More research is needed to determine if low copper levels induce the expression of these genes. It will also be important in future studies to determine whether differences in Reg mRNA levels are associated with changes in Reg protein levels, especially considering that Reg mRNA expression was quite variable within the iron-deficient and iron-overload groups. Lastly, given the exocrine and endocrine nature of the pancreas, interpretation of the microarray data would be enhanced by knowing how the iron status of pancreatic regions/cell types was affected by the different diets. In the present study, however, we were unable to histochemically detect non-heme iron in our pancreas samples because they were below the limit of detection, even by using the highly sensitive diaminobenzidine (DAB)-enhanced Perls' stain.

In conclusion, our microarray analysis of rat pancreas has revealed that iron deficiency and overload increase the expression of one or more genes strongly associated with diabetes and pancreatic stress, thus highlighting the importance of iron status in the pancreas.

## Supporting Information

Table S1 **Primers for quantitative reverse transcriptase PCR.**
(XLSX)

Table S2 **Functional categories of pancreatic genes differentially expressed in response to iron.**
(XLSX)

Table S3 **Functional categories of pancreatic genes differentially expressed in response to iron overload.**
(XLSX)
